# With peppermints you’re not my prince: Aroma modulates self-other integration

**DOI:** 10.3758/s13414-015-0955-9

**Published:** 2015-07-15

**Authors:** Roberta Sellaro, Bernhard Hommel, Claudia Rossi Paccani, Lorenza S. Colzato

**Affiliations:** Institute for Psychological Research and Leiden Institute for Brain and Cognition, Leiden University, Leiden, The Netherlands; Cognitive Psychology Unit, Leiden University, Wassenaarseweg 52, 2333 AK Leiden, The Netherlands

**Keywords:** Joint Simon effect, Self-other integration, Aromas, Cognitive state, Attention

## Abstract

Recent studies showed that self-other integration, as indexed by the joint Simon effect (JSE), can be modulated by biasing participants towards particular (integrative vs. exclusive) cognitive-control states. Interestingly, there is evidence suggesting that such control states can be induced by particular odors: stimulating odors (e.g., peppermint aroma) seem to induce a more focused, exclusive state; relaxing odors (e.g., lavender aroma) are thought to induce a broader, more integrative state. In the present study, we tested the possible impact of peppermint and lavender aromas on self-other integration. Pairs of participants performed the joint Simon task in an either peppermint- or lavender-scented testing room. Results showed that both aromas modulated the size of the JSE, although they had a dissociable effect on reaction times (RTs) and percentage of errors (PEs). Whilst the JSE in RTs was found to be less pronounced in the peppermint group, compared to the lavender and no-aroma groups, the JSE in PEs was significantly more pronounced in the lavender group, compared to the peppermint and no-aroma group. These results are consistent with the emerging literature suggesting that the degree of self-other integration does not reflect a trait but a particular cognitive state, which can be biased towards excluding or integrating the other in one’s self-representation.

## Introduction

Converging evidence suggests that the way people represent (construe) themselves is very flexible and context-sensitive, especially with regard to the degree they perceive themselves as being dependent on, or independent from, their social environment (for a review, see Cross, Hardin, & Gercek-Swing, 2011). For instance, the degree of inclusion of others into a person’s self-concept does not only vary with psychological and cultural variables (Markus & Kitayama, 1991; Triandis, 1989), but also depends on situational and contextual factors, such as the degree to which a task draws attention to one's social relatedness (Kühnen & Oyserman, 2002). This seems to reflect a tendency to adapt one’s own self-construal to the situation at hand, which again may explain why people experience a certain variation in their self-construal, in which the boundaries between oneself and others can change.

How can self-representations be so dynamic? According to Hommel, Colzato, and van den Wildenberg (2009; see also Dolk et al., 2013, 2014; cf. Hommel, Müsseler, Aschersleben & Prinz, 2001), our cognitive system represents individuals (i.e., social events) and objects (i.e., non-social events) in equivalent ways, namely, as an integrated network of codes (i.e., so-called event files) that store information about an event’s perceptual features and actions. This implies that there is no actual difference between representing oneself and representing another person, as well as between representing an individual and representing an object, as these representations rely on a common format. Accordingly, there would be no reason to assume that constructing and handling representations of oneself and of others is any different from constructing and handling representations of objects: if objects can be perceived as being more similar and related (e.g., forming a Gestalt or group) or more dissimilar and separate, depending on the context (Olson, 1970), the same should apply to people. Going a step further, this suggests that the process of integrating or discriminating between self and other can be controlled by the same mechanisms and according to the same principles that allow one to integrate or discriminate between two objects.

Empirical evidence in favor of this claim comes from recent studies showing that performance in the joint Simon task (Sebanz, Knoblich, & Prinz, 2003), which has been assumed to reflect the degree of self-other integration, is sensitive to manipulations that are likely to affect the exclusiveness versus integrativeness of cognitive control states.

In the joint Simon task, pairs of participants take turns in performing complementary parts of a Simon task (Simon & Small, 1969). For example, if the standard two-choice version of a Simon task requires pressing a left and right response key to the blue and green color, respectively, of a lateralized stimulus, the joint version would require one participant to press the left key whenever a blue stimulus occurs and the other participant to press the right key whenever a green stimulus is presented. If such a Go-Nogo version of the Simon task is carried out alone, the Simon effect (faster and/or more accurate responses if stimulus location and response location correspond) disappears (Hommel, 1996; Sebanz et al., 2003, 2006; Tsai et al., 2006; Vlainic et al., 2010; Welsh et al., 2007), suggesting that participants no longer code their single response as left or right. If the other key is operated by another individual, however, as in the joint task version, the Simon effect is back – spatial stimulus-response correspondence improves performance – the joint Simon effect (JSE; Sebanz et al., 2003). This suggests that, in joint Simon tasks, the actor takes into consideration the co-actor’s action in the spatial coding of his/her own response. According to the referential coding account (Dolk et al., 2013, 2014)^1^ spatial response coding is accomplished to solve an action discrimination problem: the spatial coding of one’s own response is functional in deciding whether a given stimulus would need to be followed by the actor’s or by the co-actor’s action (see Ansorge & Wühr, 2004, for a response-discrimination account of the Simon effect). Crucially, following this account, such a discrimination problem would be more pronounced the more the actor and the co-actor are perceived as similar.

Even though the JSE can be obtained with non-human co-actors and non-social salient events as well (Dolk, Hommel, Colzato, Schütz-Bosbach, Prinz, & Liepelt, 2011; Dolk et al., 2013), the size of the JSE has been shown to be sensitive to social factors that increase (vs. decrease) the perceived or “real” interpersonal similarity, such as the agenthood and human-likeness of the “co-actor” (Müller et al., 2011a; 2011b; Stenzel et al., 2012, 2014; Tsai & Brass, 2007; Tsai, Kuo, Hung, & Tzeng, 2008;), the quality (Hommel et al., 2009), and the nature (cooperative vs. competitive) of the personal relationship between actor and co-actor (Ruys & Aarts, 2010; Iani et al., 2011), and the collectivistic attitude of the participants (Colzato et al., 2012b). This suggests that the size of the JSE is a good indicator for the degree to which people integrate others into their self-concept.

Importantly for the purpose of the current study, other findings show that the size of the JSE can be increased by pushing participants towards a cognitive control state that favors information integration (Colzato et al., 2012a, 2013; Kuhbandner et al., 2010). For instance, Colzato et al. (2012a) had participants perform a joint Simon task after having carried out an unrelated paper-and-pencil task requiring them to circle either relational/interdependent pronouns (e.g., “we,” “our,” “us”) or independent pronouns (e.g., “I,” “my,” “me”) to induce opposite cognitive control states: a context-dependent, integrative state or a context-independent, exclusive state, respectively (cf. Kühnen & Oyserman, 2002). It is well known that cognitive control states tend to outlive the particular task or condition they have been established for and can thus bias cognitive control in a subsequent unrelated task (Allport, Styles, & Hsieh, 1994; Memelink & Hommel, 2006, 2013). By exploiting this property, Colzato et al. were able to show that inducing a context-dependent, integrative state induced by relational-pronouns circling increases the size of the JSE. Similar results were observed in a follow-up study using a similar priming procedure (Colzato et al., 2013). In this study, participants performed a joint Simon task that was interleaved with a task requiring either divergent thinking (Guilford, 1967), which requires a more distributed, integrative control mode, or convergent thinking (Mednick, 1962), which requires a more focused, exclusive control mode (Fischer & Hommel, 2012; Hommel, 2012). As was expected, the JSE was larger in the context of the divergent-thinking task than in the context of the convergent-thinking task. In a similar vein, Kuhbandner et al. (2010) showed that the induction of positive or negative mood by exposing participants to an emotionally charged movie affected performance in a subsequent joint Simon task: participants showed a larger JSE after having watched a happiness-inducing movie – a finding that fits with the assumption that positive mood induces a more integrating cognitive-control style (Ashby, Isen & Turken, 1999; Hommel, 2012).

Taken together, these findings suggest that it is possible to make people more or less integrative and, hence, to promote or to prevent self-other integration by priming an individual’s cognitive control state towards one or the other pole of the underlying control dimension. Here we investigated whether self-other integration can also be modulated by environmental factors that are likely to impact cognitive-control states. Among these factors, ambient odors (i.e., aromas) have been found to bias an individual’s attention towards either global or local representational levels (for reviews, see Herz, 2009; Johnson, 2011). Specifically, it has been suggested that stimulating aromas, such as peppermint (Barker et al., 2003; Colzato et al., 2014; Ho & Spence, 2005; Kovar et al., 1987; Moss et al., 2008; Warm et al., 1991; Raudenbush et al., 2001; Raudenbush et al., 2009; Warm and Dember, 1990), lead to a more focused, exclusive attentional state, whereas relaxing aromas, such as lavender (Basevitch et al., 2011; Diego et al., 1998; Field et al., 2005; Lehrner et al., 2005; Grimes, 1999; Guéguen & Petr, 2006; Moss, Cook, Wesnes, & Duckett, 2003; Sakamoto et al., 2005; Sellaro et al., 2015b) induce a broader, inclusive attentional state. For instance, research has found that being exposed to peppermint aroma improves memory (Moss et al., 2008), sustained visual attention (Warm et al., 1991), dual-task performance (Ho & Spence, 2005), athletic task performance (Raudenbush et al., 2001), and alertness in a driving simulator task (Raudenbush et al., 2009), and affects the allocation of attention in time (Colzato et al., 2014). In contrast, being exposed to lavender aroma has been found to lessen fatigue (Sakamoto et al., 2005), to promote behavior commitment (Grimes, 1999), to increase the amount of time customers spend in a restaurant and the amount of purchasing (Guéguen & Petr, 2006), and to enhance interpersonal trust (Sellaro et al., 2015b). Based on these premises, it should be possible to systematically bias participants towards a lesser or greater degree of self-other integration by exposing them to particular aromas. We investigated this possibility by having one group of participants perform a joint Simon task in a peppermint-scented room, and another group of participants perform the same task in a lavender-scented room. As a control condition, a third group of participants was required to carry out the joint Simon task while being exposed to no aroma. If being exposed to stimulating odors induces a more focused, exclusive control state, while being exposed to relaxing odors induces a broader, integrative state, the JSE should be affected differently by the two aromas. Specifically, whilst peppermint is expected to reduce the size of the JSE, lavender is expected to increase it.

Given that the JSE has been found to be more pronounced when consciously experiencing a positive mood (Kuhbandner et al., 2010), and that pleasant odors can increase mood (Herz, 2009), we also assessed participants’ subjective affective states, and we did so before and after the joint Simon task. To this end, we used the Affect Grid (Russell et al., 1989), a single-item scale requiring participants to rate their mood on a 9 × 9 grid, where the horizontal axis stands for affective valence (unpleasantness – pleasantness), and the vertical axis for perceived activation (high arousal – sleepiness).

## Experiment 1

### Method

#### Participants

Seventy-two healthy students of the Leiden University (mean age = 20.19 years, SD = 2.6; 16 males) participated in the experiment for partial fulfillment of course credit or a financial reward (€3). Participants were screened via a phone call by the experimenter before inclusion, using the Mini International Neuropsychiatric Interview (M.I.N.I.; Sheehan et al., 1998). The M.I.N.I. is a short, structured, interview of about 15 min that screens for several psychiatric disorders and drug use, often used in clinical and pharmacological research (Colzato & Hommel, 2008; Colzato et al., 2009; Sheehan et al., 1998). All participants were naïve regarding the purpose of the experiment and none of them reported any sensory deficits. Participants were recruited via an on-line recruiting system and came to the laboratory as unacquainted couples.^2^ Participants were equally distributed over three experimental groups: 24 participants were exposed to lavender aroma, 24 participants to peppermint aroma, and 24 participants were exposed to no aroma.

Written informed consent was obtained from all participants after a detailed explanation of the study procedures. The protocol was approved by the local ethics committee (Leiden University, Faculty of Social and Behavioral Sciences).

#### Apparatus and stimuli

The experiment was controlled by a Switch computer attached to a Philips 17-in monitor. In the joint Simon task participants made speeded discriminative responses to the (green or blue) color of circles by pressing one of two response keys of a QWERTY keyboard; the other key was operated by another participant. Circles (diameter of 43 pixels) were equiprobably presented to the left or right (at a distance of 50 pixels) of a central fixation point (12 pixels) until the response was given or 1,500 ms had passed. Intervals between subsequent stimuli varied randomly, but equiprobably, from 1750–2250 ms in steps of 100 ms. Participants were to ignore the location of the stimulus and to base their response exclusively on its color. Responses were to be given as fast and as accurate as possible; feedback was provided at the end of a trial block. The task consisted of one 60-trial practice block and three 60-trial experimental blocks. In half of the trials, stimulus and response positions corresponded (spatial stimulus-response Correspondence), whereas in the other half, stimulus and response positions did not correspond (spatial stimulus-response Noncorrespondence).

#### Procedure and design

Before and after performing the joint Simon task participants were asked to rate their mood on a 9 × 9 Pleasure × Arousal grid (i.e, the Affect Grid; Russell et al. 1989) with values ranging from –4 to 4. After the first rating, participants performed the joint Simon task. “De Tuinen™” pure essential oils (De Tuinen Aromatherapie) of peppermint and lavender were used to produce the ambient aromas. Following Colzato et al. (2014) and Sellaro et al. (2015b), four drops of the appropriate oil were applied to a candle diffuser, diluted in 30 ml of water. Two separate diffusers were used for spreading the two aromas. The diffuser was out of participants’ sight and the candle was switched on 20 min before the testing session started.

#### Statistical analysis

A significance level of *p* < .05 was adopted for all tests. Mean correct reaction times (RTs) and error percentages (PEs) were analyzed by means of repeated-measures analyses of variance (ANOVAs) as a function of Aroma Group (lavender vs. peppermint vs. control) as between-participants factor and spatial stimulus-response Correspondence (correspondence vs. noncorrespondence) as within-participants factor. Pleasure and Arousal scales were analyzed separately by means of two repeated-measures ANOVAs with time (first vs. second measurement) as a within-participants factor and condition (lavender vs. peppermint vs. control) as a between-participants factor. Fisher least significance difference (LSD) post-hoc tests were performed to clarify mean differences in case of significant interactions.

### Results

#### Joint Simon task

**Fig. 1:**
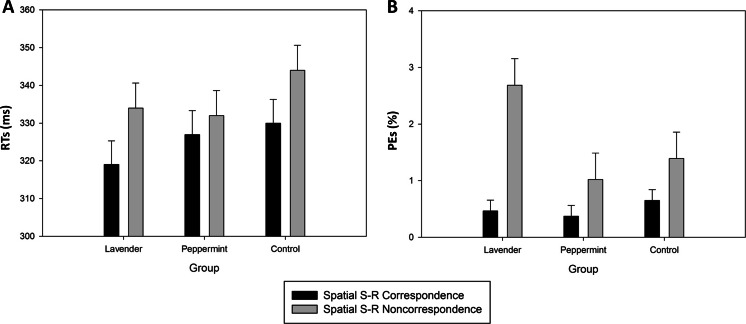
Experiment 1 (joint Simon task): Mean correct reaction times (RTs; panel **a**) and percentage of errors (PEs; panel **b**) as a function of group (lavender, peppermint, and control) and spatial stimulus-response (S-R) correspondence. Error bars show standard errors of the means

The RTs analysis yielded a main effect of Correspondence, *F*(1,69) = 52.698, *p* < .0001, MSE=97.446, *η*^*2*^_*p*_=0.43, indicating that responses were faster with spatial S-R correspondence than with noncorrespondence (325 vs. 337 ms). This effect was modified by a significant interaction involving Aroma Group, *F*(2,69)=3.789, *p*<.05, MSE=97.446, *η*^*2*^_*p*_=0.10. Fisher’s LSD post-hoc tests showed no differences between groups when comparing RTs on spatial S-R correspondence [*p*_*s*_≥.14; 95 % CI_(control vs. lavender)_ = (−7.0, 29.4); 95 % CI_(control vs. peppermint)_ = (−15.0, 21.4); 95 % CI_(lavender vs. peppermint)_ = (−26.2, 10.2)], nor when comparing RTs on spatial S-R noncorrespondence [*p*_*s*_≥.19; 95 % CI_(control vs. lavender)_ = (−8.2, 28.2); 95 % CI_(control vs. peppermint)_ = (−6.0, 30.4); 95 % CI_(lavender vs. peppermint)_ = (−16.0, 20.4)]. However, post-hoc tests did reveal that the correspondence effect differed between groups, with the difference between spatial S-R correspondence and noncorrespondence (i.e., the JSE) being significant for the lavender [16 ms; *p*<.01; 95 % CI_(correspondence vs. noncorrespondence)_ = (−21.4, −10.0)] and control [15 ms; *p*<.001; 95 % CI_(correspondence vs. noncorrespondence)_ = (−20.2, −8.8)] groups, but not for the peppermint group [6 ms; *p*=.054; 95 % CI_(correspondence vs. noncorrespondence)_ = (−11.3, 0.1)] (see Fig. 1, panel A). As expected, the size of the JSE was significantly less pronounced in the peppermint group as compared to both lavender [*p*<.05; 95 % CI_(lavender vs. peppermint)_ = (2.1, 18.2)] and control [*p*<.05; 95 % CI_(control vs. peppermint)_ = (0.9, 17.0)] groups, which showed comparable sizes [*p*=.77; 95 % CI_(control vs. lavender)_ = (−9.2, 6.9)].

The main effect of Aroma Group was not significant, *F*(2,69)<1, p=.47.

The PEs analysis revealed a main effect of Correspondence, showing that fewer errors were made in correspondence trials (0.5 %) than in noncorresponding trials (1.7 %), *F*(1,69)=19.106, *p*<.0001, MSE=2.730, *η*^*2*^_*p*_=0.22. This effect was modified by Aroma Group, *F*(2,69)=3.429, *p*<.05, MSE=2.730, *η*^*2*^_*p*_=0.09. Fisher’s LSD post-hoc tests revealed that PEs were comparable across groups on spatial S-R correspondence [*p*_*s*_≥.58; 95 % CI_(control vs. lavender)_ = (−1, 1); 95 % CI_(control vs. peppermint)_ = (−0.8, 1.2); 95 % CI_(lavender vs. peppermint)_ = (−0.9, 1.1)], but not on spatial S-R noncorrespondence. Specifically, on noncorresponding trials participants in the lavender group produced significantly more errors than participants in the control [*p*<.05; 95 % CI_(control vs. lavender)_ = (−2.3, −0.3)] and peppermint [*p*<.005; 95 % CI_(lavender vs. peppermint)_ = (0.7, 2.7)] groups, who were comparable [*p*=.47; 95 % CI_(control vs. peppermint)_ = (−0.6, 1.4)]. The correspondence effect differed between groups, with the JSE in terms of PEs being significant for the lavender group [2.2 %; *p*<.001; 95 % CI_(correspondence vs. noncorrespondence)_ = (−3.2, −1.3)], but not for the control [0.7 %; *p*=.12; 95 % CI_(correspondence vs. noncorrespondence)_ = (−1.7, 0.2)] and peppermint [0.6 %; *p*=.18; 95 % CI_(correspondence vs. noncorrespondence)_ = (−1.6, 0.3)] groups. The JSE in PEs was significantly more pronounced in the lavender group as compared to both peppermint [*p*<.05; 95 % CI_(lavender vs. peppermint)_ = (0.2, 2.9)], and control [*p*<.05; 95 % CI_(control vs. lavender)_ = (−2.8, −0.1)] groups, which showed comparable sizes [*p*=.89; 95 % CI_(control vs. peppermint)_ = (−1.3, 1.4)] (see Fig. 1, panel B). The main effect of Aroma Group was not significant, *F*=2.786, *p*=.07.

#### Mood

ANOVAs performed on the Pleasure (1.2 vs. 1.3 in the peppermint group, 1.0 vs. 1.0 in the lavender group, and 1.5 vs. 1.4 in the control group) and Arousal (.7 vs. .8 in the peppermint group, −.3 vs. −.2 in the lavender group, and .3 vs. −.1 in the control group) scales revealed no main effects or interactions, *F*_*s*_≤2.717, *p*_*s*_≥.07. This suggests that we can rule out an account of our results in terms of (conscious) pleasure and arousal changes.

### Discussion

As expected, the size of the JSE was found to be affected by the specific aroma participants were exposed to while performing the joint Simon task. Indeed, we observed that the JSE in RTs was significantly less pronounced in the peppermint group compared to both lavender and no-aroma groups. The smaller JSE found in the peppermint group is consistent with the hypothesis that being exposed to stimulating odors, like peppermint, favors a more focused, exclusive control mode such to emphasize the distinction between self and other, by drawing attention to local details (i.e., details that make one person different from another, e.g., dress, hair color). As the size of the JSE varies as a function of the degree of similarity between self and other (e.g., Hommel et al., 2009), focusing attention on local details reduced its size accordingly. In contrast with our expectations, however, the exposure to lavender aroma did not significantly increase the size of the JSE in RTs compared to the group of participants who was not exposed to any aroma. As previously mentioned, being exposed to relaxing odors, like lavender, was expected to favor a broader, more inclusive cognitive control mode such as to increase self-other overlap by drawing attention to global representational levels (i.e., to the commonalities, e.g., the fact that you and I are both humans). The failure to observe a modulation of the JSE in RTs via lavender exposure might be taken to suggest that the selected aroma was ineffective in inducing a more integrative cognitive-control state to affect the size of the JSE accordingly. However, as the size of the JSE is typically very small, the lack of a lavender modulation might also be due to a ceiling effect. This alternative possibility is supported by the fact that, when looking at the JSE in terms of errors, we found that lavender, compared to peppermint and no aroma, gave rise to a more pronounced effect, suggesting that being exposed to lavender did in fact impact performance.

Taken together, the present findings support the hypothesis that being exposed to ambient odors that are suspected to impact differentially one’s own cognitive-control state can affect the degree of self-other integration (i.e., the JSE) accordingly. However, given that in a joint Simon task the actions of a co-actor do not play any role in one’s own performance, taking these actions into consideration (i.e., showing a JSE) can be seen as a failure to exclude irrelevant information from processing. Accordingly, it is possible that the exposure to peppermint and lavender aromas affected attentional control processes rather than the degree of self-other integration. Specifically, the smaller JSE in the peppermint group might reflect improved attentional control rather than reduced self-other integration. Likewise, the larger JSE in the lavender group found in terms of errors might be due to a lack of attentional control (i.e., increased distraction). To rule out this possibility we ran a second experiment where three new groups of participants were confronted on a standard two-choice Simon task while being exposed to peppermint, lavender, or no-aroma. Given that in this task stimulus position is irrelevant (Hommel, 2011), increased attentional control – possibly caused by peppermint exposure – would be expected to reduce the size of the Simon effect by limiting the impact of stimulus position on response selection. By comparison, a lack of attentional control – possibly induced by lavender exposure – would be expected to produce a larger Simon effect by increasing the impact of stimulus position on response selection. Should we fail to observe any modulation of the size of the standard Simon effect, this would suggest that, in Experiment 1, aromas affected specifically self-other integration.

## Experiment 2

### Method

#### Participants

Seventy-two healthy students of the Leiden University (mean age = 20.90 years, SD = 3.5; four males) participated in the experiment for partial fulfillment of course credit or a financial reward (€3). As in Experiment 1, all participants were prescreened by a phone interview using the M.I.N.I. (Sheehan et al., 1998). Participants were not aware of the purpose of the experiment, they did not participate in the previous experiment, and they did not report any sensory deficits. Participants were tested individually and were equally distributed over three experimental groups: 24 participants were exposed to lavender aroma, 24 participants to peppermint aroma, and 24 participants were exposed to no aroma.

All participants gave their written informed consent. The protocol was approved by the local ethics committee (Leiden University, Faculty of Social and Behavioral Sciences).

#### Apparatus, stimuli, and procedure

The apparatus, stimuli, and procedure were as in Experiment 1 with the following exceptions. All participants were tested individually and performed a two-choice Simon task requiring them to operate both response keys: the left key in response to the green circle and the right key in response to the blue circle. The task comprised one 60-trial practice block and two 60-trial experimental blocks, half with spatial S-R correspondence and half with spatial S-R noncorrespondence.

RT, PE, and mood data were analyzed as in Experiment 1.

### Results

#### Simon task

**Fig. 2:**
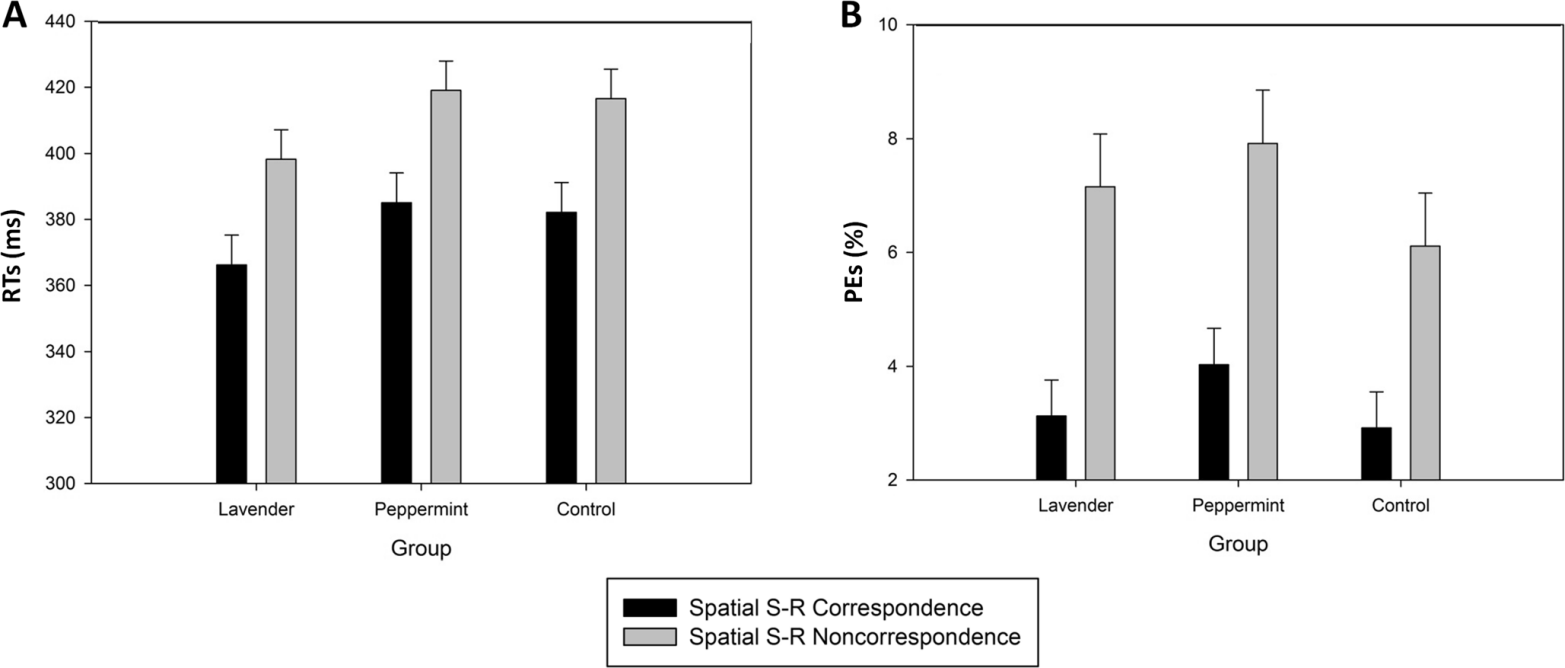
Experiment 2 (two-choice Simon task): Mean correct reaction times (RTs; panel **a**) and percentage of errors (PEs; panel **b**) as a function of group (lavender, peppermint, and control) and spatial stimulus-response (S-R) correspondence. Error bars show standard errors of the means

The RT and PE analyses revealed significant main effects of Correspondence, *F*(1,69) = 190.014, *p* < .0001, MSE=212.42, *η*^*2*^_*p*_=0.73 (RT), *F*(1,69) = 44.525, *p* < .0001, MSE=11.09, *η*^*2*^_*p*_=0.39 (PE). Participants were faster and produced less errors on corresponding (378 ms and 3.4 %, respectively) than on noncorresponding (411 ms and 7.1 %) trials. Importantly, neither the main effects of Aroma Group nor the interactions involving Correspondence and Aroma Group were significant, *F*_*s*_≤1.52, *p*_*s*_≥.23 (see Fig. 2, panels A and B).

#### Mood

ANOVAs performed on the Pleasure (1.0 vs. 0.7 in the peppermint group, 0.9 vs. 1.0 in the lavender group, and 0.8 vs. 0.8 in the control group) and Arousal (1.0 vs. 0.8 in the peppermint group, 0.8 vs. 1.0 in the lavender group, and 0.7 vs. 0.8 in the control group) scales revealed no main effects nor interactions, F_s_<1, p_s_≥.45.

### Discussion

Unlike what we found in Experiment 1, the size of the Simon effect was not significantly modulated by the specific aroma participants were exposed to. Indeed, the three experimental groups showed comparable standard Simon effects in both RTs and PEs. This suggests that the effect of aromas found in Experiment 1 was specific to the JSE and, thus, affected self-other integration rather than attentional control processes.

## General discussion

In the present study we exposed participants to either a stimulating aroma (i.e., peppermint) or a relaxing aroma (i.e., lavender) to induce a more focused, exclusive or a more distributed, integrative cognitive control state, respectively. If successful, this should have affected the degree of self-other integration, as assessed by the size of the JSE. Results showed that the size of the JSE in RTs, but not in PEs, was less pronounced in the group who performed the joint Simon task in the peppermint-scented laboratory as compared to the groups who performed the task in the lavender-scented and no-scented laboratories. By comparison, being exposed to lavender, compared to the peppermint and control (no aroma) conditions, increased the size of the JSE in PEs, but not in RTs. This suggests that both selected aromas affected the degree of self-other integration, although they had a dissociable effect on RTs and PEs. Importantly, the fact that either aromas did not affect the size of the standard Simon effect (Experiment 2) indicates that the effect of aroma was specific to the JSE and, thus, undermines an interpretation of these results in terms of attentional control changes. The present findings suggest that aromas can act as a cognitive modulator, favoring either an exclusive or an integrative control mode. We propose that the more exclusive state induced by peppermint modulates information processing in such a way that attention is drawn to the local details, thus emphasizing the distinction between self and other (Hommel et al., 2009). Conversely, when a more inclusive state is induced by lavender and/or by typical experimental settings (here, no-aroma condition) local details tend to be ignored (or weighted less; see Memelink & Hommel, 2013) while attention is drawn to the global stimulus situation – the “big picture.” As a consequence, the representations of self and other become less distinct and overlap more (Hommel et al., 2009), so that self and other are perceived more as parts of a common whole. According to the referential coding account (Dolk et al., 2013, 2014), this makes response discrimination more difficult, which again makes participants attend the most salient response-discriminating feature: response location. Increasing the weight of the response-location code increases the feature overlap with the stimuli, which increases the size of the stimulus-response correspondence effect.

Interestingly, we did not observe any evidence that pleasure and/or arousal levels mediated the observed outcome. However, our measures relied on conscious self-report and thus reflect merely conscious aspects of the participant’s affective state. We thus cannot rule out the possible impact of more implicit pleasure and arousal changes that future studies might assess by including physiological measurements, such as galvanic skin response, heart rate, and diastolic and systolic blood pressure.

The present study has some limitations that need to be considered. First, we did not verify whether participants were aware of the presence of the aroma, whether they could recognize the specific aroma they were exposed to, and whether they perceived peppermint and lavender as really arousing and relaxing, respectively. Second, we did not assess participants’ olfactory sensitivity. Thus, we cannot be confident that all participants were able to perceive the scents. Therefore, future studies should extend our findings by including self-report ratings of the sprinkled scents, as well as standardized tests to assess participants’ olfactory threshold.

From a broader perspective, our present results are consistent with, and complement, previous findings suggesting that self-other integration does not reflect a trait but, rather, is the consequence of a particular, temporary cognitive control state. As this control state can apparently be affected by particular odors, our observations suggest that the social attitude of people can be effectively modulated by suitable scents: cooperation would be likely to benefit from relaxing scents while competition should benefit from stimulating scents. Moreover, given that people do not depend on odors to implement a particular control state, they could also actively prepare for collaborative or competitive social challenges by relaxing or stimulating themselves, respectively. In any case, our observations suggest that social attitudes are and can be controlled by the same mechanisms and according to the same principles that allow one to control other cognitive operations (Colzato et al., 2013, 2012a, 2012b; Hommel et al., 2009).

## Footnotes

1 It is worth mentioning that since the seminal study of Sebanz et al. (2003), different interpretations have been advanced to explain the occurrence of the JSE. According to the original interpretation – the action/task corepresentation account (Knoblich & Sebanz, 2006; Sebanz & Knoblich, 2009) – this effect is due to the automatic co-representation of another human beings (i.e., the other’s actions and/or the task rules and intentions that govern them) through a dedicated social action-perception mechanism. However, this interpretation has been undermined by more recent findings showing that this effect often reflects the influence of spatial factors inherent in the task (Dittrich, Rothe, & Klauer, 2012; Dittrich, Dolk, Rothe-Wulf, Klauer, & Prinz, 2013; Guagnano, Rusconi, & Umilta, 2010; Sellaro et al., 2013), is not specific to the situation involving human co-actors (Dolk et al., 2011; 2013), and is highly sensitive to the temporary-induced cognitive control state (Colzato et al., 2013, 2012a; Kuhbandner et al., 2010). Taken together these findings made it necessary to reconsider the assumed social nature of the effect and, accordingly, alternative non-social interpretations were proposed: the spatial response coding account (Dittrich, et al., 2012, 2013; Guagnano, et al., 2010), which considers the JSE as a purely spatial phenomenon, and the referential coding account (Dolk et al., 2011, 2013, 2014; see text for more details), according to which this effect relies on universal information processing mechanisms (for a detailed description of the differences between these two account, see Dolk et al., 2014, and Sellaro et al., 2015a).

2 Participants who did not show up were replaced by a confederate in the joint task. Participants were not aware that their partner was a confederate.
